# Platelet-derived microvesicles induce calcium oscillations and promote VSMC migration *via* TRPV4

**DOI:** 10.7150/thno.47182

**Published:** 2021-01-01

**Authors:** Shan-Shan Li, Shuang Gao, Yi Chen, Han Bao, Zi-Tong Li, Qing-Ping Yao, Ji-Ting Liu, Yingxiao Wang, Ying-Xin Qi

**Affiliations:** 1 Institute of Mechanobiology& Medical Engineering, School of Life Sciences &Biotechnology, Shanghai Jiao Tong University, Shanghai, China; 2 School of Perfume and Aroma Technology, Shanghai Institute of Technology, Shanghai, China; 3 Department of Bioengineering, Institute of Engineering in Medicine, University of California, San Diego, San Diego, United States; 4 Key Laboratory for Biomechanics and Mechanobiology of Ministry of Education, School of Biological Science and Medical Engineering, Beihang University, Beijing, 100083, China; 5 Beijing Advanced Innovation Center for Biomedical Engineering, Beihang University, Beijing, 100083, China

## Abstract

**Rationale:** Abnormal migration of vascular smooth muscle cells (VSMCs) from the media to the interior is a critical process during the intimal restenosis caused by vascular injury. Here, we determined the role of platelet-derived microvesicles (PMVs) released by activated platelets in VSMC migration.

**Methods:** A percutaneous transluminal angioplasty balloon dilatation catheter was used to establish vascular intimal injury. Collagen I was used to activate PMVs, mimicking collagen exposure during intimal injury. To determine the effects of PMVs on VSMC migration *in vitro*, scratch wound healing assays were performed. Fluorescence resonance energy transfer was used to detect variations of calcium dynamics in VSMCs.

**Results:** Morphological results showed that neointimal hyperplasia was markedly increased after balloon injury of the carotid artery in rats, and the main component was VSMCs. PMVs significantly promoted single cell migration and wound closure *in vitro*. Fluorescence resonance energy transfer revealed that PMVs induced temporal and dynamic calcium oscillations in the cytoplasms of VSMCs. The influx of extracellular calcium, but not calcium from intracellular stores, was involved in the process described above. The channel antagonist GSK219 and specific siRNA revealed that a membrane calcium channel, transient receptor potential vanilloid 4 (TRPV4), participated in the calcium oscillations and VSMC migration induced by PMVs.

**Conclusions:** TRPV4 participated in the calcium oscillations and VSMC migration induced by PMVs. PMVs and the related molecules might be novel therapeutic targets for vascular remodeling during vascular injury.

## Introduction

Vascular intimal injury occurs following cardiovascular disease treatments, such as bypass surgery, coronary vein graft, angioplasty and stent treatment 1. As a consequence, intimal hyperplasia, even postangioplasty restenosis, may contribute to treatment failure 2,3. Vascular smooth muscle cells (VSMCs), the dominant cellular component of arteries, are primarily present within the media layer of vessels under physiological conditions. However, it has been well established that abnormal VSMC media-to-intima migration is an important cellular process during intimal hyperplasia 4-6. Therefore, studying the mechanism of abnormal VSMC migration during intimal hyperplasia is of great significance for improving the cure rate of cardiovascular disease.

Adult VSMCs retain the potential to alter their migrative properties, and this process can be regulated by extracellular matrix components, peptide growth factors, cytokines, RNA molecules, mechanical factors, ion signaling and other environmental cues 7-9. For example, metalloproteinase-2 (MMP2) and metalloproteinase-9 (MMP9) participate in VSMC migration from the media to the intima following arterial injury and alter postinjury vascular remodeling 10,11. Wu et al. revealed that kindlin-2 plays a critical role in VSMC proliferation, migration and intimal hyperplasia *via* Wnt signaling, and blocking the activity of kindlin-2 is an attractive therapeutic approach for vascular injury 12. Blood flow and shear stress have also been found to abrogate the proliferative and migratory response of VSMCs in the early stages after injury 13,14. In addition to these regulatory responses, numerous studies have revealed that the regulation of cell migration is critically dependent on calcium. Chemoattractants stimulate neutrophil migration by inducing repeated transient increases in intracellular calcium level 15. Excitatory neurotransmitters initiate cell contraction, which is the key process in cell migration, by interacting with cell surface receptors to generate inositol 1,4,5-trisphosphate (IP_3_) 16. IP_3_ binds to inositol 1,4,5-trisphosphate receptors (IP_3_Rs) on the sarcoplasmic reticulum to trigger calcium release, which sustains elevated levels of cytoplasmic calcium to regulate colonic smooth muscle contraction.

Although the studies described above have revealed many important factors that affect VSMC migration, the underlying mechanisms during intimal hyperplasia are still not fully understood. During vascular intimal injury, the adherence and accumulation of circulating platelets to the injured intima is an important pathological process 17,18. Circulating platelets can be activated by exposure to collagen (mainly collagen I), which is caused by the injury of monolayer endothelial cells (ECs). Activated platelets can release a variety of heterogeneous platelet-derived microvesicles (PMVs) 17, which are mainly 100-1000 nm in diameter 19-21. PMVs are capable of selectively carrying different types of biomolecules, such as membrane and cytoplasm proteins, lipids, RNAs, and other bioactive molecules, and transferring these biomolecules to recipient cells, thus participating in the regulation of recipient cell functions 17,18,22. It has been reported that thrombin/collagen-induced PMVs can enhance the potential of early outgrowth cells (EOCs) to restore endothelial integrity by transferring chemokine receptor-4 (CXCR4) to EOCs after vascular injury 23.

PMVs have been reported to play critical roles in various cardiovascular diseases, including hypertension, atherosclerosis and thrombin formation 18,24. Under conditions of intimal injury, the internal elastic lamina can be broken, and PMVs can thus directly contact the middle layer 25. Even under conditions of mild intimal injury, the EC layer can be broken, and the fenestrae on the internal elastic lamina (1~3 µm in width) 26 can allow PMVs (10~1000 nm in diameter) to diffuse into the middle layer. However, the relationship between PMVs and VSMC migration during intimal hyperplasia remains poorly characterized. In this study, collagen I was used to activate PMVs, mimicking collagen exposure during intimal injury, and the roles of PMVs in VSMC migration were demonstrated. Our bioinformatic analysis, which was based on previously published proteomic data of collagen-induced PMVs 27, suggested that calcium may be the key node in the regulatory network of PMV-induced cell migration. We investigated the calcium dynamics induced by PMVs in live VMSCs at the single-cell and subcellular levels by using fluorescence resonance energy transfer (FRET) combined with a calcium biosensor. We then further identified the potential calcium channels that participate in PMV-induced calcium oscillations in VSMCs.

## Materials and Methods

### Animal model

The animal care and experimental protocols were conducted in accordance with the Animal Management Rules of China (55, 2001, Ministry of Health, China), and the study was approved by the Animal Research Committee of Shanghai Jiao Tong University.

Male Sprague Dawley rats were housed in a temperature-controlled room with a 12-h light/dark cycle and were given access to standard chow and tap water ad libitum. Vascular injury was established in 8-week-old rats, as previously described 3. Briefly, a percutaneous transluminal angioplasty balloon dilatation catheter (Boston Scientific Corporation, Galway, Ireland) was used to establish vascular intimal injury. All the animals were anesthetized by isoflurane inhalation and treated under sterile conditions. The balloon was placed into the left carotid artery and repeatedly stretched to damage the blood vessels. The undamaged right carotid artery served as the self-control 3.

### HE and immunofluorescence staining

Four weeks after balloon injury, the arteries were removed, fixed in 4% paraformaldehyde solution, embedded, and finally cut into 8-μm sections. HE staining 28 or immunofluorescence staining 29 was performed as previously described. For immunofluorescence staining, the paraffin-embedded sections were permeabilized with 0.1% Triton X-100 for 10 min. After treatment with phosphate-buffered saline (PBS) containing 10% goat serum for 1 h, the sections were coincubated with primary rabbit anti-von Willebrand Factor antibody (1:200, Dako, Copenhagen, Denmark A/S) and mouse monoclonal anti-α-SMA antibody (1:200, Dako, Copenhagen, Denmark A/S) for 24 h at 4°C. Secondary anti-mouse/rabbit IgG antibodies (1:1000, Cell Signaling Technology, Boston, MA) were used. The nuclei were stained with DAPI after immunofluorescence staining. The fluorescence images were acquired by using a fluorescence microscope (Olympus, Tokyo, Japan).

### PMV isolation, collection and size analysis

PMVs were isolated according to the previous literatures 30,31. As shown in Supplementary Figure 1A, whole blood was collected from the abdominal aorta of anesthetized rats into a syringe containing 100 μL/mL anticoagulant in 0.5% sodium chloride solution. The blood was centrifuged at 1500 rpm for 10 min to obtain platelet-rich plasma and then centrifuged at 2800 rpm for 15 min to obtain platelets. The platelets were resuspended using Tyrode solution and activated with collagen I at 37°C. After activating for 90 min, the solution was centrifuged at 2800 rpm for 15 min to remove the platelets, and the PMVs were collected at 20500 g/min for 90 min. The PMVs were analyzed with a flow cytometry column (FACSCalibur TM; BD Biosciences) combined with a platelet-specific marker (anti-rat CD41) to demonstrate the platelet origin^30^,^31^, and with a NanoSight3000 high-sensitivity detection system (Malvern Panalytical, Malvern, England) to analyze the particle size.

### PMV treatment

After the carotid artery balloon injury model was established, PMVs (6×10^9^ per ml) or normal saline (as a control group) were injected into the tail veins of the rats on alternate days for 2 weeks after the surgery. On day 14 after the surgery, the injured carotid artery was collected.

### Cell culture and transfection

VSMCs were isolated from the thoracic aortas of male Sprague Dawley rats *via* an explant method, as previously described 32. The VSMCs were cultured in Dulbecco's modified Eagle's medium (DMEM) (GIBCO, NY, USA) with 10% calf serum (FCS, GIBCO, NY, USA), 2 mM glutamine (GIBCO, NY, USA), 100 U/mL penicillin and 100 μg/mL streptomycin (BBI Company, Shanghai, China) at 37°C in a 5% CO_2_ atmosphere. The VSMCs were characterized by antibody specific for α-smooth muscle actin (1:1000, Dako, Copenhagen, Denmark A/S). In all the experiments, the purity of the cell populations was universally higher than 95%, and only VSMCs between passages 4 and 7 were used.

For the transfection process, a FRET-based calcium biosensor, which includes a pair of fluorescent proteins (ECFP and YPet) and calmodulin-linked light chain protein kinase M13, was utilized. The structure of the biosensor is altered after binding to calcium ions 33. Adenovirus vectors (Genepharma, Shanghai, China) were used to introduce the reconstructed plasmid containing the FRET-based calcium biosensor into VSMCs.

### Microscopy, image acquisition and analysis

Cells were starved in DMEM without FCS for 6 h before PMV stimulation. Then, PMVs were gently added to the culture dish. During the imaging experiments, the cells were maintained in streptomycin-free medium to prevent possible effects on calcium ion channels. All the images were obtained by using a Leica inverted microscope (DMi8, Wetzlar, Germany) equipped with a charge-coupled device (CCD) camera (Andor iXon 897, Belfast, UK) and two emission filters controlled by a filter changer (480DF20 for ECFP and 535DF15 for YPet). During the image capturing process, a temperature-control system with CO_2_ supplement was used to maintain cellular viability. Time-lapsed fluorescence images were acquired at 30-s intervals by Leica LASX software (Leica Biosystems GmbH). The emission ratio of FRET/ECFP was directly computed and generated by the Leica LASX software before being subjected to quantification and analysis. The max ratio and calcium oscillation frequency were further analyzed.

### Scratch wound healing assay

To assess VSMC migration *in vitro*, scratch wound healing assays were performed. A wound healing cell migration assay was performed using 95% confluent cells, as described in previous studies 34,35. A line was scratched across a monolayer of cells using a sterile 10-μL pipette tip. Images of the scratched line were immediately captured, and the cells were imaged again after 12 h and 24 h (4X objective, IX-71, Olympus, Japan). The wound area was measured using ImageJ software (NIH, USA). The cell migration abilities were calculated as (S_0_ - S_t_)/ S_0_, where S_0_ is the wound area at the initial time point, and S_t_ is the wound area at the observation time point t (12 h or 24 h).

### Solutions and chemicals

For the experiments that required calcium-free conditions, calcium-free DMEM (GIBCO, NY, USA) was used. The chemical reagents 2-aminoethoxydiphenyl borate (2-APB) (0.1 mM) 36 and nifedipine (10 μM) 37 were purchased from Sigma-Aldrich (St. Louis, MO). GSK219 (0.1 mM) 38 was obtained from Merck (NY, USA). All the drugs were preincubated with VSMCs for 20 min before the addition of the PMVs. The amount of drug administered was based on previous publications.

### RNA interference

For RNA interference, VSMCs were transfected with 50 nM small interfering RNA (siRNA) fragments (si-1/2/3) or control nonsilencing siRNA (si-NC) (Genepharma, Shanghai, China) for 24 h using Lipofectamine 2000 (Invitrogen, Carlsbad, CA) according to the manufacturer's instructions 39. The sequences of the small interfering RNA fragments targeting TRPV4 (sequence accession number NM_023970.1) and si-NC are listed in Table S1.

### Western blot

Lysates were separated by 10% SDS-PAGE. The proteins were detected using primary antibodies against TRPV4 (1:500, Alomone Labs, Jerusalem, Israel) and GAPDH (1:1000, Protein tech, Beijing, China). An HRP-labeled IgG was used as the secondary antibody at a 1:1000 dilution, and the bands were visualized using an ECL kit (Beyotime, Shanghai, China) and quantified with Quantity One software (Bio-Rad, Hercules, CA, USA).

### Statistical analysis

All the values are expressed as the mean ± standard error (S.E.M.) of the mean, and all the data are consistent with variance after R studio software. The statistical analysis of the data was performed by unpaired Student's t-test to identify significant differences between two mean values, and a value of P < 0.05 was considered statistically significant. The statistical analysis was performed using Excel (Microsoft Corporation, Washington, USA) and GraphPad 9.0 (GraphPad Software, San Diego, CA).

## Results

### Vascular intimal injury promotes VSMC migration and neointimal hyperplasia

**Figure 1:**
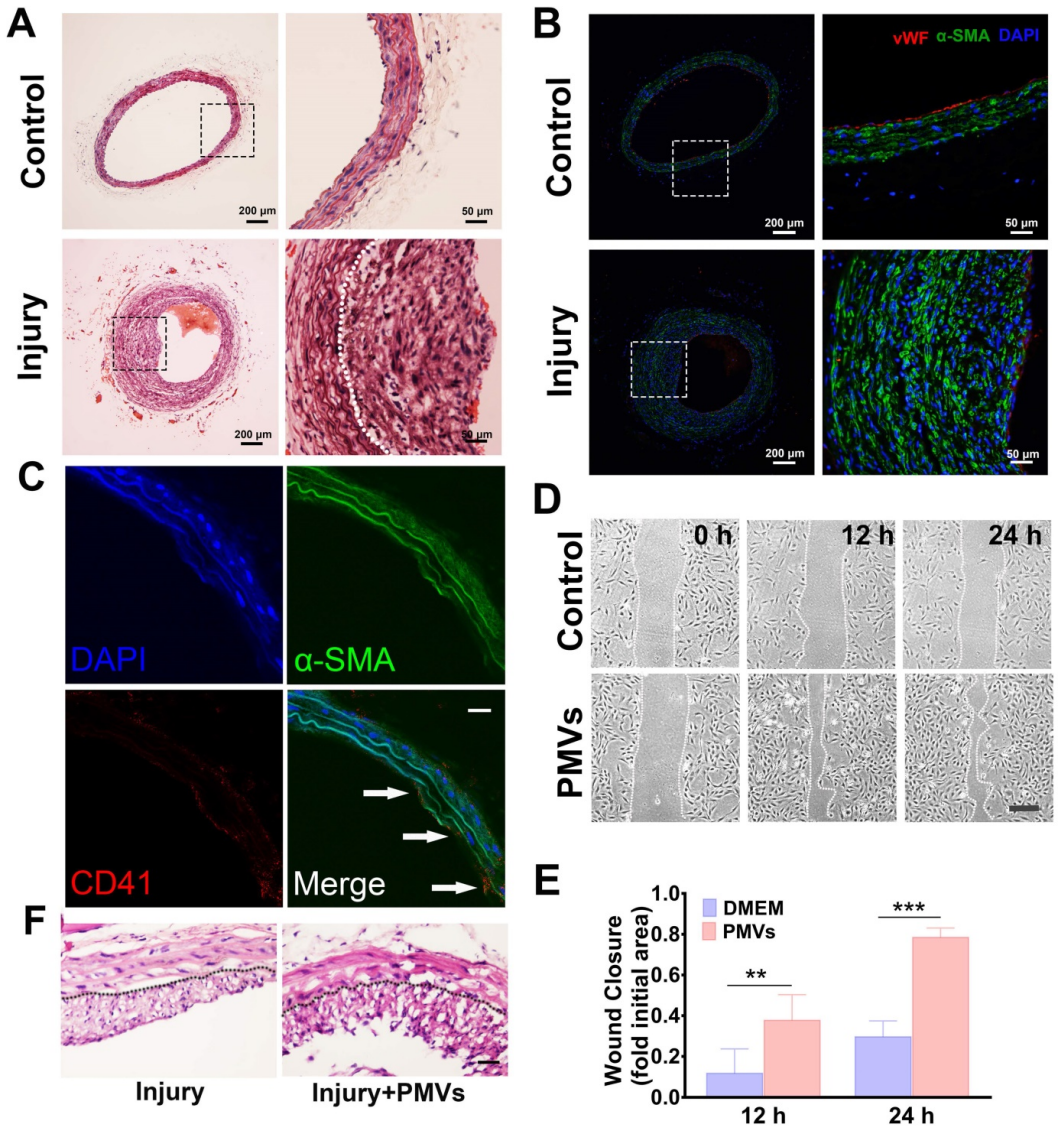
Increased VSMC migration after intimal injury *in vivo* and PMV treatment *in vitro* and *in vivo*. (A) HE staining revealed that the neointima was markedly thickened 4 weeks after vascular intimal injury compared with the self-artery control (control). The white dotted line represents the inner elastic fibers. Scale bar: 200 μm (left panel); 50 μm (right panel). (B) Immunofluorescence staining revealed that neointimal hyperplasia mainly consists of VSMCs. VSMCs were identified by α-SMA (green), and ECs were identified by von Willebrand factor (vWF, red). vWF, a marker of ECs, was discontinuously expressed on the lumen of damaged blood vessels in the intimal injury models (bottom panel). Cell nuclei were stained with DAPI (blue). Scale bar: 200 μm (left panel); 50 μm (right panel). (C) PMVs adhered to VSMCs in neointimal hyperplasia *in vivo*. Twenty-four hours after injury, carotid arteries were collected and then examined by immunofluorescence staining. PMVs were identified by CD41 (red), and VSMCs were identified by α-SMA (green). Cell nuclei were stained with DAPI (blue). The white arrows indicate typical locations where PMVs adhere to VSMCs. Scale bar: 50 μm. (D) The migration of VSMCs stimulated with DMEM control or PMVs for 12 h/24 h, as detected by the wound healing assay. Scale bar: 200 μm. (E) The histogram shows the fold change in the level of VSMC migration stimulated by PMVs (n = 8) relative to that stimulated by the DMEM control (n = 7). The values are shown as the mean ± S.E.M. for each condition. ** P < 0.01, *** P < 0.001. (F) PMVs promote neointimal hyperplasia in injured carotid arteries on Day 14 after surgery. Scale bar: 100 μm.

Four weeks after intimal injury, HE staining revealed that compared with that of the self-contralateral noninjured artery, the neointimal layer of the intimal injured artery was markedly thickened, and the area of the vascular cavity was markedly reduced (Figure 1A). In addition, immunofluorescence staining revealed the expression of α-smooth muscle actin (α-SMA) in neointimal hyperplasia, which suggested that the main component of the vascular neointima was VSMCs (Figure 1B). During vascular intimal injury, collagen exposure activates platelets, which accumulate and release a variety of heterogeneous vesicles, including PMVs. *In vivo* immunofluorescence staining for CD41 (a marker of PMVs 40) and α-SMA (a marker of VSMCs 41) showed that CD41-positive particles were closely adjacent to α-SMA-positive cells, indicating that PMVs accumulate at the injured artery and contact VSMCs *in vivo* (Figure 1C). We then demonstrated the role of PMVs in VSMC migration and the potential mechanisms.

After using collagen I to simulate the pathological exposure of extracellular matrix components during intimal injury, PMVs were extracted, and the diameter of the PMVs was further examined by NanoSight3000. The results illustrated that the sizes of the PMVs were distributed within a range of 100~600 nm, and there were 3 peaks at 121.6 nm, 183.0 nm and 291.5 nm (Figure S1).

PMVs can adhere to VSMCs *in vitro* (Figure S2A). Our scratch wound data showed that PMVs induced a marked increase in the wound repair capabilities of VSMCs at 12 h and 24 h (Figure 1D). The averaged wound closure areas at 24 h are 78.42% and 29.64% smaller than the initial area at 0 h for the PMVs group and the control, respectively (Figure 1E). Moreover, the track results of the free migration of single cells revealed that compared with the control (Figure S2B, Video S1A), PMV treatment increased the migration distance of single VSMCs in a time-dependent manner, and a significant increase was detected at approximately 10.5 h (Figure S2B, Video S1B).

These results suggested that the PMVs released by collagen I-activated platelets promote the migration of VSMCs, which may participate in the intimal hyperplasia caused by intimal injury *in vivo*. We further conducted *in vivo* experiments to investigate PMV-induced neointimal hyperplasia in arteries. First, a carotid artery balloon injury model was established. Then, PMVs (6×10^9^ per 1 mL) or normal saline (as a control group) were injected through the tail veins of the rats on alternate days for 2 weeks after the surgery. Fourteen days after the surgery, the injured carotid artery was collected. Neointimal hyperplasia was examined with HE staining. As shown in Figure 1F, the thickness of the neointimal layer of the rats was considerably increased by PMVs.

### PMVs induce calcium oscillations in VSMCs

**Figure 2:**
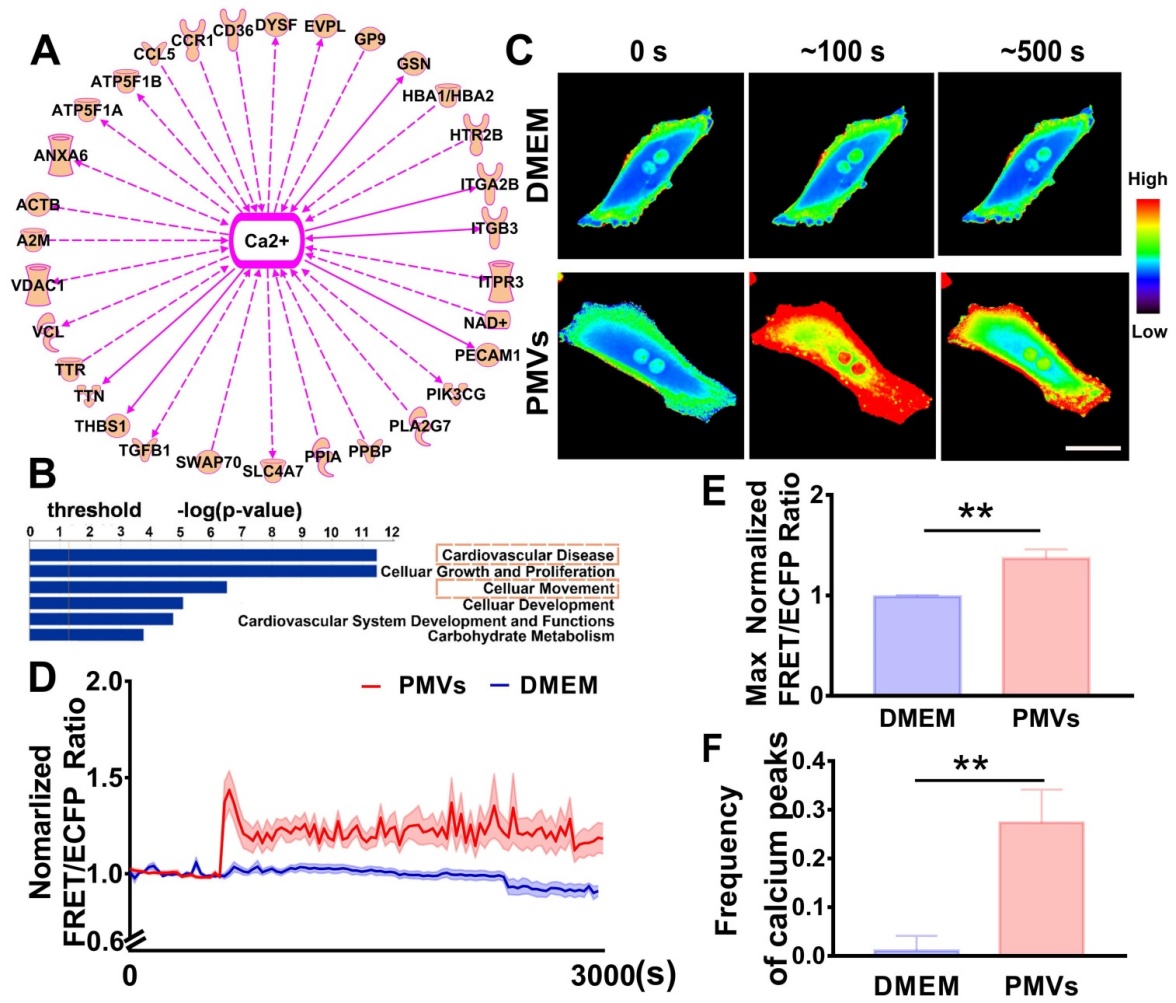
Intercellular calcium oscillations in VSMCs in response to PMVs. (A) The proteomic data were analyzed by IPA, which revealed that calcium signaling may be the key node in regulating cell migration. (B) Bar graphs demonstrate the top cell and disease functions regulated by PMV-expressed proteins. (C) Time-lapse FRET images of changes in cytoplasmic calcium in VSMCs treated with PMVs and the DMEM control. The hot and cold colors represent high and low FRET ratios, indicating high and low levels of cytoplasmic calcium change, respectively. Scale bar: 30 μm. (D) The time courses represent the normalized FRET/ECFP ratio averaged over the cell body in VSMCs treated with PMVs (n = 23) and DMEM (n = 10), and all shadowed areas indicate the S.E.M. Comparison of the max normalized FRET/ECFP ratio (E) and frequency of cytoplasmic calcium oscillations (F) between VSMCs treated with PMVs (n = 23) and those treated with DMEM (n = 10). The data are expressed as the mean ± S.E.M. ** P < 0.01.

Based on previously published proteomic data 27, IPA software was used to analyze the potential mechanism by which PMVs participate in VSMC migration. The results revealed that 31 proteins expressed in PMVs were correlated with calcium signaling (Figure 2A, Table S2), and cellular movement was one of the critical identified functions that are regulated by PMVs (Figure 2B). Then, the spatiotemporal characteristics of the calcium dynamics in VSMCs treated with PMVs were characterized with the aid of FRET real-time microscopy.

The representative heat map of the FRET/ECFP ratio showed that compared with the DMEM control, PMVs triggered marked increases in the calcium levels in live VSMCs (Figure 2C, Video S2A-B). The average FRET/ECFP ratio increased by ~1.5-fold within 60 s and fluctuated at high levels for a period of 3000 s in VSMCs treated with PMVs (Figure 2D). Furthermore, the maximum ratio of FRET/ECFP in response to PMV treatment also significantly increased (1.3646 ± 0.0934 *vs.* 0.9869 ± 0.0149) (Figure 2E), and the frequency of calcium peaks upon PMV stimulation was significantly increased with those induced by the DMEM control (0.2733 ± 0.0681 *vs.* 0.0105 ± 0.0307) (Figure 2F). Emission spectral analysis showed that after PMV treatment, the ECFP emission peak at 475 nm decreased, and the YPet emission peak at 525 nm increased. The results revealed that PMVs enhanced YPet emission at the expense of ECFP emission, indicating a calcium-induced gain of FRET (Figure S3).

Calcium peaks that increase in amplitude modulation and frequency modulation are defined as calcium oscillations 42, and these results suggested that PMVs induced calcium oscillations in target VSMCs. There are two main sources of cytosolic calcium, the influx of calcium from the extracellular matrix and the release of calcium from intracellular stores; thus, the role of these two sources in PMV-induced calcium oscillation were explored in further studies.

### Extracellular calcium participates in calcium oscillations and VSMC migration

**Figure 3:**
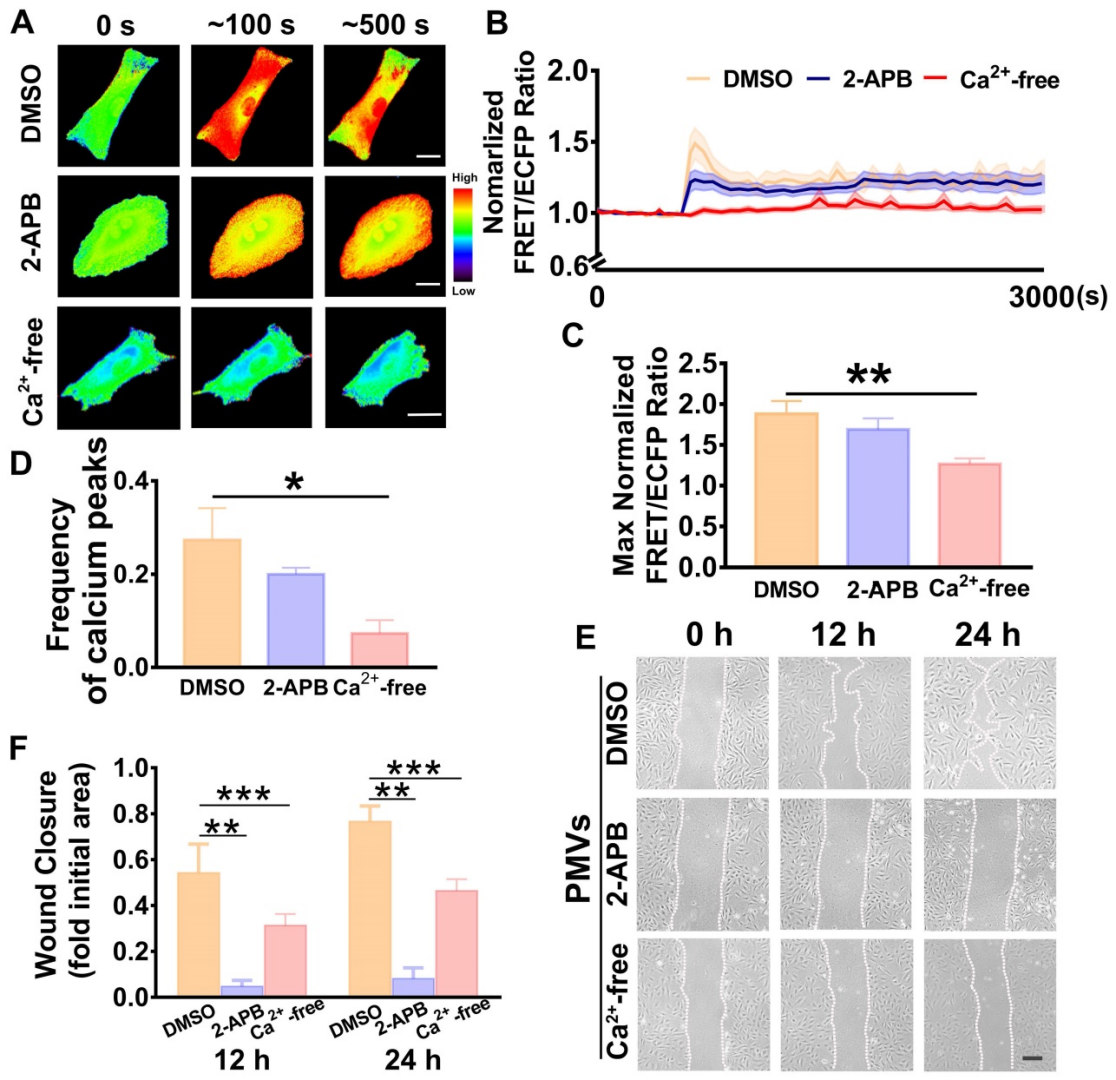
PMV-induced calcium oscillations in VSMCs and wound closure in calcium-free medium or 2-APB-containing medium. (A) FRET images of calcium upon PMV treatment of VSMCs pretreated with DMSO control (top), 2-APB (middle) and calcium-free medium (bottom). The hot and cold colors represent high and low FRET ratios, indicating high and low levels of cytoplasmic calcium change, respectively. Scale bar: 30 μm. (B) The time courses represent the normalized FRET/ECFP ratio averaged over the cell body in VSMCs pretreated with 2-APB (n = 14, blue), calcium-free medium (n = 19, red) and DMSO control (n = 10, orange) after PMV treatment, and all shadowed areas indicate the S.E.M. Comparation of the max normalized FRET/ECFP ratio (C) and frequency of cytoplasmic calcium oscillations (D) among VSMCs pretreated with 2-APB (n = 14, blue), calcium-free medium (n = 19, red) and DMSO control (n = 10, orange) after PMV treatment. (E) The migration of VSMCs pretreated with DMSO control (top), 2-APB (middle) and calcium-free medium (bottom) after PMV treatment. Scale bar: 200 μm. (F) The histogram shows the fold change in the level of 2-APB- and calcium-free medium-pretreated VSMC migration relative to the DMSO control-pretreated VSMC migration. The values are shown as the mean ± S.E.M. for each condition (n = 6). * *P < 0.05,* ** *P < 0.01,* *** *P < 0.001.*

To identify the calcium source, calcium-free medium was used to remove the extracellular calcium. The results showed that the calcium oscillations induced by PMV treatment were immediately abrogated in the calcium-free medium (Figure 3A, Video S3A), and the time-lapsed FRET/ECFP ratio line was horizonal and steady throughout the time course (Figure 3B). Compared with the DMSO control, PMV treatment led to significantly decreased frequencies of calcium peaks (0.0733 ± 0.0281 *vs.* 0.2733 ± 0.0681) and max FRET/ECFPs ratios (1.265 ± 0.0690 *vs* 1.8810 ± 0.1580) in the calcium-free medium (Figure 3C-D). Moreover, the wound healing assay revealed that the calcium-free medium also markedly reversed the migration of VSMCs induced by PMVs at 12 h and 24 h (Figure 3E-F).

Binding of IP_3_ to IP_3_Rs on the sarcoplasmic reticulum triggers the release of intracellular calcium 16. Hence, 2-APB, an antagonist of IP_3_Rs 36, was used to block the release of calcium from intracellular stores. The representative FRET/ECFP ratio maps are shown in Figure 3A (Video S3B-C). There was no significant difference in the average FRET/ECFP ratio (Figure 3B), the frequency of calcium peaks (0.2000 ± 0.014) (Figure 3C) or the max ratio (1.691 ± 0.1347) (Figure 3D) of the calcium dynamics between the 2-APB and DMSO control groups (Figure 3C-D).

These results suggested that oscillations were diminished by calcium-free medium, indicating that the influx of extracellular calcium was an important source of the calcium oscillations in the VSMCs.

### TRPV4 channel, but not L-type voltage-gated calcium channel, mediates PMV-induced calcium oscillations

Since transient receptor potential vanilloid 4 (TRPV4) and L-type voltage-dependent calcium channel (L-VDCC) are widely reported to be abundantly expressed in VSMCs and related to important functions 42-44, the possible roles of these two channels that regulate the influx of extracellular calcium were further demonstrated.

**Figure 4:**
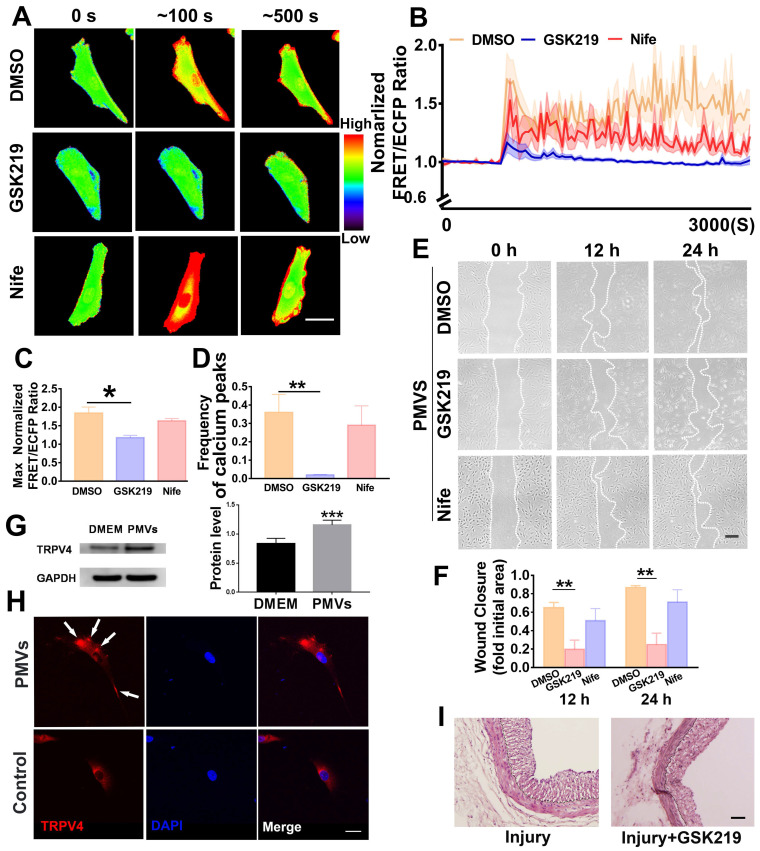
The PMV-induced calcium oscillations in VSMCs and wound closure are abolished by the TRPV4 antagonist GSK219 but not by Nife. (A) The color images represent the time-lapse FRET images of the changes in cytoplasmic calcium upon PMV treatment of VSMCs pretreated with GSK219 (middle), Nife (bottom) and the DMSO control (top). The hot and cold colors represent high and low FRET ratios, indicating high and low levels of cytoplasmic calcium change, respectively. Scale bar: 30 μm. (B) The time courses represent the normalized FRET/ECFP ratio averaged over the cell body in VSMCs pretreated with GSK219 (n = 19, blue), Nife (n = 8, red) and DMSO (n = 12, orange) after PMV treatment, and all shadowed areas indicate the S.E.M. Comparation of the max normalized FRET/ECFP ratio (C) and frequency of cytoplasmic calcium oscillations (D) among VSMCs pretreated with GSK219 (n = 19, blue), Nife (n = 8, red) and DMSO control (n = 12, orange) after PMV treatment. (E) The migration of VSMCs pretreated with GSK219 (middle), Nife (bottom) and DMSO control (top) after PMV treatment. Scale bar: 200 μm. (F) The histogram shows the fold change in the level of GSK219- and Nife-pretreated VSMC migration relative to the that of DMSO control-pretreated VSMC migration. The values are shown as the mean ± S.E.M. for each condition (n = 6). * *P < 0.05,* ** *P < 0.01*. (G) The protein level of TRPV4 was increased after PMV treatment for 24 h. *** *P* < 0.001 *vs.* DMEM control (n = 5). (H) Immunofluorescence staining of TRPV4 (red) and nuclei (blue) of VSMCs after treatment with PMVs (top panel) or the DMEM control (bottom panel) for 5 min. Arrows indicate typical regions of dense and punctate TRPV4 staining. Scale bar: 40 μm. (I) TRPV4 inhibitor GSK219 attenuated neointimal hyperplasia in injured carotid arteries on Day 28 after the surgery. Scale bar: 50 μm.

GSK219 (GSK2193874, 0.1 mM), the specific antagonist of TRPV4, significantly reduced the calcium oscillations in response to PMV treatment (Figure 4A-D, Video S4B). Compared with the DMSO solvent control, GSK219 significantly decreased the max ratio of FRET/ECFP and the frequency of VSMC calcium peaks, and these values were 1.1770 ± 0.0635 *vs.* 1.8420 ± 0.1642 and 0.02 ± 0.0013 *vs.* 0.36 ± 0.098, respectively (Figure 4C-D, Video S4A-B). VSMC wound closure was suppressed at 12 h and 24 h by preincubation with GSK219 (Figure 4F). Nifedipine (Nife), the specific chemical inhibitor of L-VDCC, was used. Compared with the DMSO solvent control (Video S4A), Nife (10 μM) had no significant effect on the calcium oscillations (Figure 4A-B, Video S4C), the max ratio of FRET/ECFP (1.6341 ± 0.0633* vs.* 1.8420 ± 0.1642) or the frequency (0.2901 ± 0.1056 *vs.* 0.36 ± 0.098) of the VSMC calcium peaks induced by PMVs. The wound closure rates induced by Nife and the solvent DMSO were also similar (Figure 4E-F).

The change in TRPV4 protein expression in VSMCs before and after PMV treatment was examined with Western blot assays. The Western blot results indicated that the protein level of TRPV4 was increased after PMV treatment for 24 h (Figure 4G). Immunofluorescence staining demonstrated that the TRPV4 ion channels were distributed more densely in punctate regions on the cell membrane after PMV treatment for 5 min (Figure 4H).

We further conducted *in vivo* experiments to investigate if GSK219 ameliorates neointimal hyperplasia in rats. GSK219 (1 mg/kg) or normal saline (as a control group) was intraperitoneally injected 1 h before carotid artery balloon injury and on day 7, day 14 and day 21 after the surgery. On Day 28 after the surgery, the injured carotid artery was collected. Neointimal hyperplasia was examined by HE staining. As shown in Figure 4I, the thickness of the neointimal layer in the rats was considerably decreased by GSK219.

These results suggested that TRPV4, a member of the transient receptor potential (TRP) family, participates in the influx of extracellular calcium induced by PMVs, which may subsequently modulate VSMC migration.

### TRPV4 knockdown abolishes calcium oscillations and cell migration

**Figure 5:**
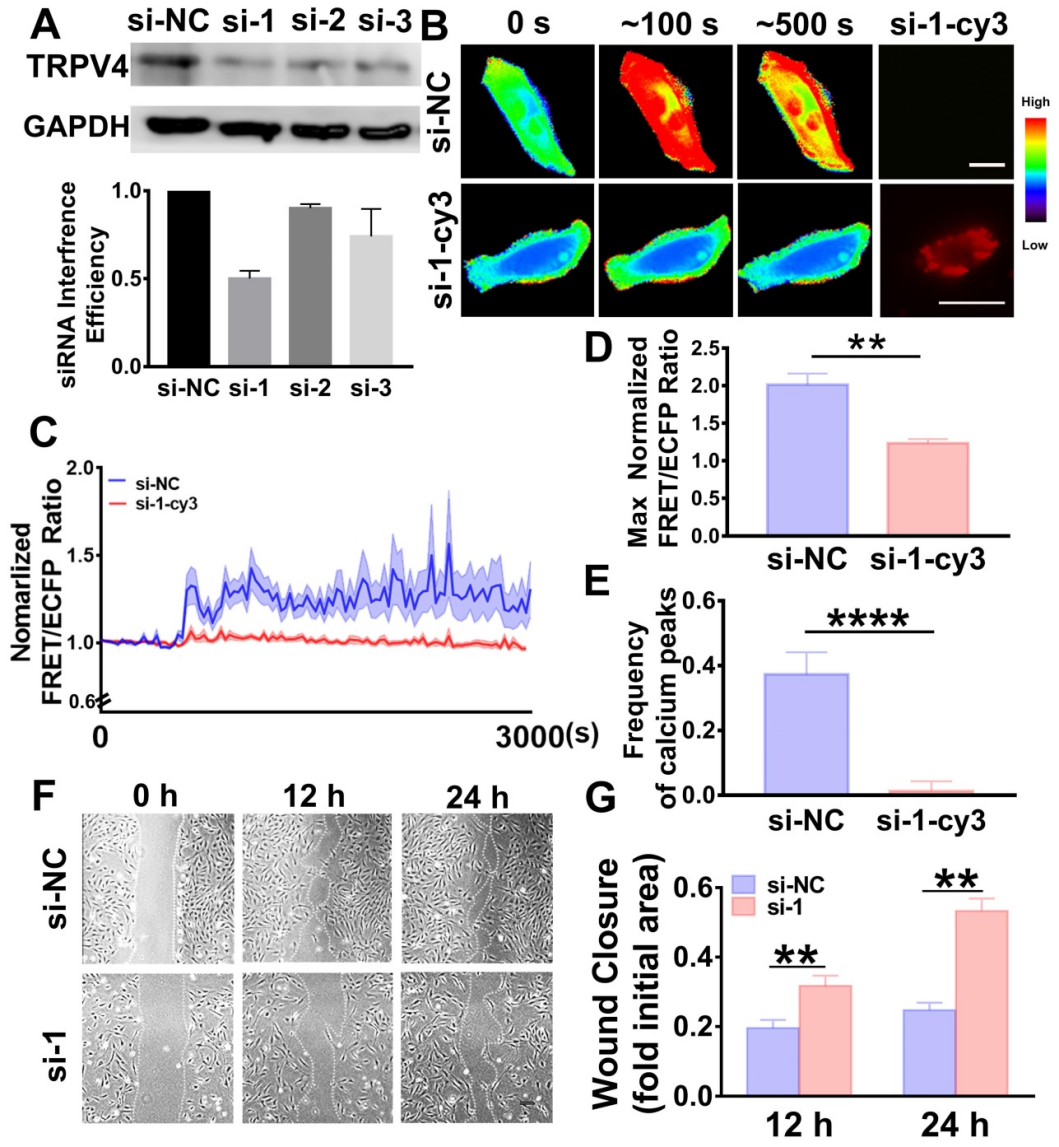
TRPV4 siRNA abolished calcium oscillations and VSMC wound closure. (A) Western blot results indicate the interference efficiencies of 3 TRPV4 siRNA sequences. (B) Time-lapse FRET images of the chances in cytoplasmic calcium in VSMCs transfected with TRPV4 si-1-Cy3 or si-NC during the PMV treatment process. The hot and cold colors represent high and low FRET ratios, indicating high and low levels of cytoplasmic calcium change, respectively. Scale bar: 30 μm. (C) The time courses represent the normalized FRET/ECFP ratio averaged over the cell body in VSMCs transfected with TRPV4 si-1-Cy3 (n = 16) or si-NC (n = 13) after PMV treatment, and all the shadowed areas indicate the S.E.M. Comparation of the max normalized FRET/ECFP ratio (D) and frequency of the cytoplasmic calcium oscillations (E) between the VSMCs transfected with TRPV4 si-1-Cy3 (n = 16) or si-NC control (n = 13) after PMV treatment. (F) The migration of VSMCs transfected with TRPV4 si-1 or si-NC control after 24 h of PMV treatment. Scale bar: 200 μm. (G) The histogram shows the fold change in the level of TRPV4 si-1-transfected VSMC migration relative to that of the si-NC control-transfected VSMC migration. The values are shown as the mean ± S.E.M. for each condition (n = 6). ** *P < 0.01,* **** P < 0.0001.

To further elucidate whether the effects of PMVs on VSMC migration and calcium oscillation were dependent on TRPV4, TRPV4 small interference RNA (siRNA) was transfected into VSMCs to knock down TRPV4. Then, FRET and wound healing were assessed. Three pairs of specific siRNAs were designed, and the most efficient siRNA, si-1, was identified (Figure 5A).

For further study, si-1 was labeled with the red fluorescent probe Cy3 (si-1-Cy3) and used to verify the effective silencing of TRPV4. Heat maps of the FRET/ECFP ratio revealed that in the si-1-Cy3 positive cells, the calcium oscillations were remarkably abolished and exhibited a steady state (Figure 5B, Video S5B); these results were not observed in the si-NC-positive cells (Figure 5B, Video S5A). After PMV treatment, the max ratio of FRET/ECFP was 1.2341 ± 0.0544 and the frequency of calcium peaks was 0.0125 ± 0.0307 in the si-1-Cy3-positive cells, indicating that these effects were all significantly abolished (Figure 5D-E); these results were not observed in the si-NC-positive cells (the max ratio was 2.0141 ± 0.1464 and the frequency was 0.3733 ± 0.06806). VSMC migration was also significantly suppressed by TRPV4 si-1 transfection (Figure 5F-G), and similar results were observed after incubation with the specific inhibitor GSK219.

Here, we validated that TRPV4 in VSMCs is essential for eliciting the calcium oscillations and VSMC migration triggered by PMVs.

## Discussion

PMVs are submicroscopic (∼<1000 nm) membrane vesicles released by platelets during activation and selectively carry different molecules, including GP IIb/IIIa, GP Ib, P-selectin, and CXCR4 45. Low concentrations of PMVs are observed in normal circulation. Highly increased concentrations of PMVs may be an important indicator of thrombosis formation 18,24, atherosclerosis, hypertension and cardiopulmonary bypass 24. PMVs have been studied in the pathogenesis of many diseases. Kim et al. reported that *in vitro*, PMVs promote human umbilical vein endothelial cell (HUVEC) survival and stimulate migration and elicit tube-like structure formation in angiogenesis 46. PMVs stimulate p42/p44 MAP kinase phosphorylation, cellular oncogene Fos (c-Fos) induction and DNA synthesis in a concentration-dependent manner to promote the proliferation and migration of coronary artery smooth muscle cells under conditions of thrombosis formation 47. However, the molecular mechanisms underlying these functions of PMVs are still largely unknown.

It has been reported that platelets, which immediately accumulate at the sites of injury once the intact endothelium is damaged during vascular injury, become activated and release PMVs 48. Our present research revealed that PMVs promote the migration of VSMCs, which may participate in neointimal hyperplasia during vascular injury, and calcium is the crucial molecule underlying this process.

Calcium is the simplest and most versatile second messenger involved in regulating various cellular functions, both under physiological and pathological conditions 49-51. The calcium levels in the cytoplasm are usually low and become significantly increased when cells respond to stimulation, such as stimulation by growth factors and mechanical factors. In the present study, the increased calcium levels have been demonstrated as calcium influx from the extracellular environment. Increased calcium levels lead to further calcium binding to calmodulin to form a complex that activates downstream pathways, such as phosphatidylinositol 3-kinase (PI_3_K), calcium-dependent protein kinases II (CaMKII), and myosin light chain kinase (MLCK) 38. As a downstream molecule of calcium, CaMKII promotes VSMC migration during vascular injury through the posttranscriptional regulation of MMP9 11,52. Increased calcium activates PI_3_K/threonine-specific protein kinase (Akt) signaling *via* calmodulin in different cell lines 53,54. In addition, MLCK causes changes in the fluorescence emission and the polarization excitation spectra of the enzyme by binding to calmodulin 55. Due to its multifaceted roles, calcium signaling is the crucial coordinator of cell migration, this regulation occurs partly through local calcium pulses activating MLCK and modulating nascent focal adhesions 56. In regulating the persistence of cell movement, calcium levels are highest at the rear and lowest at the leading edge of cells 57. Previous literatures have demonstrated that extracellular calcium influx can active small GTPases Cdc42 58, Rac1 59 and RhoA 60, and thus promote cell migration. Here we found that the protein levels of all these three molecules were significantly increased by the PMVs (Figure S5), which suggested that extracellular calcium influx induced by PMVs may regulate VSMC migration *via* small GTPases.

The concentration and real-time distribution of calcium can be visualized at the molecular level in single live cells. In our present study, a calcium biosensor based on FRET was used to detect the calcium dynamics induced by PMVs. The genetically encoded calcium biosensor based on FRET consists of a pair of fluorescent indicators [enhanced cyan fluorescent protein (ECFP) and a yield YFP for energy transfer (YPet)] and calmodulin-linked light chain protein kinase M13, and this biosensor has been shown to be a useful tool to characterize intracellular cytoplasmic calcium in live cells 63. Upon increased binding of free calcium to calmodulin-linked light chain protein kinase M13, conformational changes lead to a decrease in the distance between the fluorescent protein pairs and an increase in FRET. The changes in the FRET/ECFP ratio are used to characterize the calcium dynamics in live cells.

VSMCs express a variety of ion channels in their cell membranes that mediate calcium influx in response to many environmental stimuli 64. However, the identity of plasma membrane-associated calcium permeable pathways has not been reported in PMV-stimulated VSMCs. Among the calcium ion channels on VSMCs, the TRPV4 channels play crucial roles in regulating cellular functions 65,66. TRPV4 channels are calcium-permeable nonselective cation channels on the cell membrane that are widely expressed in the cardiovascular system, including on endothelial cells, cardiac fibroblasts, and VSMCs 67. In the present study, TRPV4 were indicated abundantly expressing in the neointimal hyperplasia (Figure S6). Hu et al. revealed that TRPV4 channels mediate the FSS-induced calcium influx and osteogenic differentiation of MSCs, and these effects were inhibited by the selective TRPV4 inhibitor HC-067047 and TRPV4-specific siRNA 32. TRPV4 is required for the transforming growth factor-β (TGF-β)-induced differentiation of cardiac fibroblasts into myofibroblasts, which is critically involved in cardiac remodeling 68. Activation of the TRPV4 channel at the plasma membrane appears to reflect the activation of existing channel structures with conformational changes within the homotetrameric structure that lead to channel opening 69. Cao et al. found that protein kinase A-mediated Ser-824 phosphorylation is required for TRPV4 activation in endothelial cells and other systems 29. Note that the 2-APB investigated in the present study (Figure 3) is a typical inhibitor of IP3R that blocks intracellular calcium release from the endoplasmic reticulum 36,63 and thus suppresses cell migration. The FRET results indicate that 2-APB does not significantly affect the PMV-induced calcium increase, which further demonstrates that the calcium oscillation in the present study mainly occurs due to the influx of extracellular calcium. To reveal the mechanism of the effect of PMVs on TRPV4, we first conducted bioinformatics analysis with IPA software (Supplement Figure S7), and the results suggested that AGTR1, P2RY1 and Pka are the most important regulatory proteins upstream of TRPV4. Based on the 457 proteins previously reported to be expressed in PMVs by Dean et al. 27, the bioinformatics analysis suggested that 31 potential molecules in PMVs may participate in calcium regulation (Supplement Table 1). We further analyzed the connections between the three regulatory proteins upstream of TRPV4 (AGTR1, P2RY1 and Pka) and molecules from PMVs. As shown in Supplement Figure S7, the results identified the following possible pathways: 1) TGFB1 in PMVs affected TRPV4 by regulating P2RY1 70, and 2) CCL5 and CD36 in PMVs affected TRPV4 by regulating Pka 71,72. The effects of microvesicles from endothelial cells and VSMCs on calcium oscillations and TRPV4 channel activation in VSMCs were examined with FRET, and the results are presented in Figure S8-S10. These results suggested that the regulatory mechanism of PMVs described above could not be the same in endothelial cell microvesicles or VSMC microvesicles. However, it still requires further studies to determine the specific stimulating composition in PMVs in order to fully understand the regulating mechanisms.

**Figure 6:**
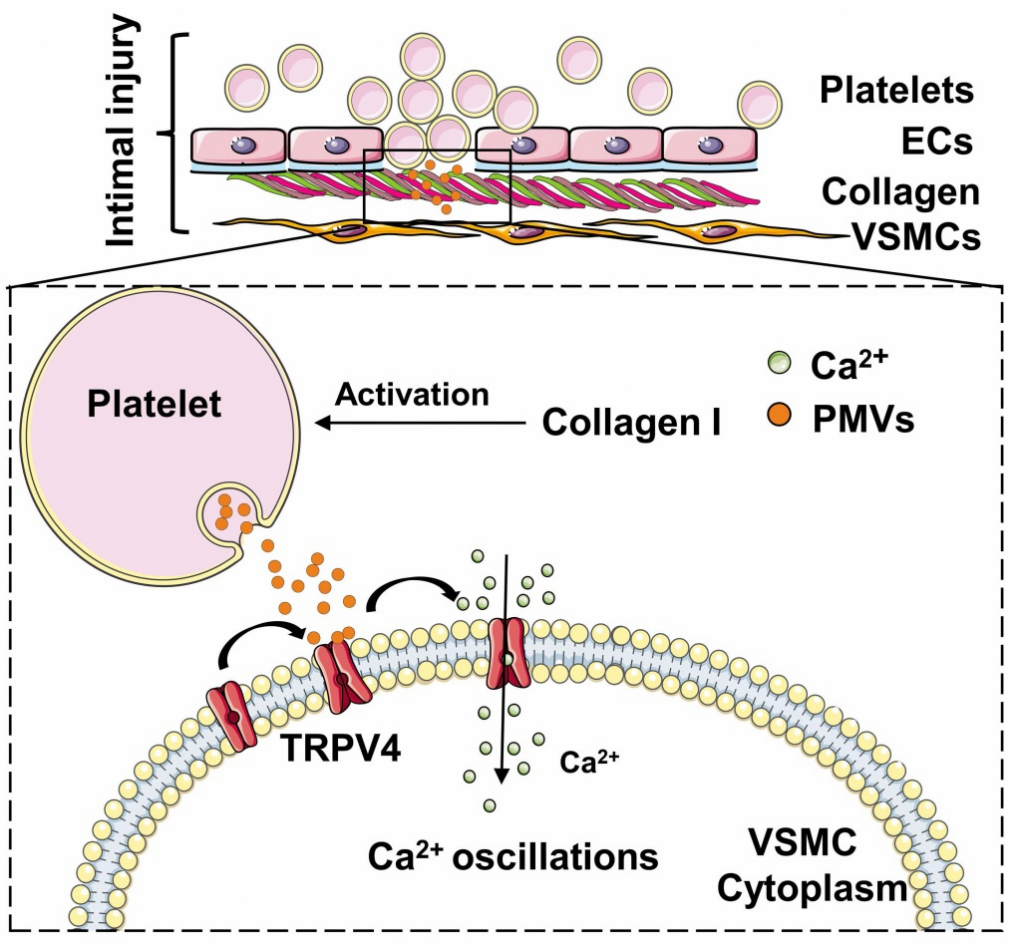
Schematic drawing of the role of the calcium oscillations induced by PMVs in VSMC migration. Collagen-induced PMVs targeted TRPV4 and induced VSMC migration.

In conclusion, our findings identified the role of PMVs, specifically those induced by collagen exposure during vascular injury, in increasing VSMC migration (Figure 6). PMVs trigger the influx of extracellular calcium *via* the TRPV4 channel, which subsequently induces calcium oscillations and promotes VSMC migration. The study may provide new insights into the mechanism of abnormal VSMC migration after intimal injury and may have potential clinical applications for attenuating neointimal hyperplasia after intimal injury during vascular stent surgery. Further studies are encouraged to reveal the influences and mechanisms of antiplatelet drugs, such as aspirin 73-75 and clopidogrel 76, in PMV-induced calcium oscillations and *in vivo* regulatory functions.

## Supplementary Material

Supplementary figures and tables.

Supplementary video 1A.

Supplementary video 1B.

Supplementary video 2A.

Supplementary video 2B.

Supplementary video 3A.

Supplementary video 3B.

Supplementary video 3C.

Supplementary video 4A.

Supplementary video 4B.

Supplementary video 4C.

Supplementary video 5A.

Supplementary video 5B.

## Abbreviation

2-APB: 2-aminoethoxydiphenyl borate
α-SMA: α-smooth muscle actin
CXCR4: chemokine receptor-4
DMEM: Dulbecco's modified Eagle's medium
ECs: endothelial cells
EOCs: early outgrowth cells
FRET: fluorescence resonance energy transfer
IP_3_: inositol 1,4,5-trisphosphate
IP_3_Rs: inositol 1,4,5-trisphosphate receptors
L-VDCC: L-type voltage-dependent calcium channel
MMP: metalloproteinase
siRNA: small interfering RNAs
PMVs: platelet-derived microvesicles
TRPV4: transient receptor potential vanilloid 4
VSMCs: vascular smooth muscle cells
