# Supplementary material for: Platelet-derived microvesicles induce calcium oscillations and promote VSMC migration via TRPV4
Source: Theranostics. 2021 Jan 1;11(5):2410–23. doi: 10.7150/thno.47182 (PMC7797689; doi:10.7150/thno.47182)
Supplement: Supplementary file 1 — Supplementary figures and tables. [file thnov11p2410s1.pdf]

47182f4

Supplemental Information

Supplement Experimental Procedures

Reaction solution component reagents

Tyrode solution:12 mM $NaHCO_3$, 138 mM NaCl, 5.5 mM glucose, 2.9 mM KCl, 2 mM $MgCl_2$, 0.42 mM NaH_2PO_4, and 10 mM 4-(2-hydroxyethyl)-1-piperazineethanesulfonic acid (pH 7.4); Anticoagulant:2.94% sodium citrate, 136 mM glucose, 0.1 g/ml PGE1, 1 U/ml apyrase (pH 6.4); Lysis buffer: 50 mM Tris-HCl, 2% (w/v) SDS, 0.1% (w/v) bromophenol, 10% (v/v) glycerol, 1% (w/v) β-mercaptoethanol (pH 6.8).

Time-lapse video microscopy

Time-lapse video microscopy was used to obtain the free migration cell tracks of individual VSMCs, as described in a previous study [1]. Briefly, the cell motility data were captured using an EVOS FL Auto Cell Imaging system (10x objective, Thermo Fisher, NY, USA). During the image capturing process, a temperature-control system with CO_2 supplement was used to maintain cell viability. Digital images were taken every 30 min for 24 h. The cell outlines and xy-centroids were determined using ImageJ software (NIH, USA).

Ingenuity pathway analysis

We applied Ingenuity Pathway Analysis (IPA) software (Qiagen) (https://www.qiagenbioinformatics.com/products/ingenuitypathway-analysis, content version: 43605602) to determine the relation among biological function, calcium molecules and PMV proteomics. IPA integrates the available knowledge on genes, drugs, chemicals, protein families, processes, and pathways based on the interactions and functions derived from the Ingenuity Pathways Knowledge Database Literature. IPA is used to understand the complex biological and

chemical systems at the core of life science research based on lectures or predicated analysis [2].

Supplement Results

We first explored the calcium effects for long time. Representative heat maps of normalized FRET/ECFP ratio at 0 s, ~500 s, ~1000 s, ~5000 s with PMVs illustrated calcium oscillations (Figure S4A). Bright field of VSMC showed a normal spreading morphology at the termination of 5000 s (Figure S4B). The average FRET/ECFP ratio were increased ~1.5-folds within 60 s with PMVs, and returned to static status ratio at about 5000 s (Figure S4C), related to Figure 2.

Supplement Reference

1. Allahverdian S, Chaabane C, Boukais K, Francis GA. Smooth muscle cell fate and plasticity in atherosclerosis. Cardiovasc Res. 2018; 114(4): 540-550.
2. Dalby B, Cates S, Harris A, Ohki EC, Tilkins ML, Price PJ, et al. Advanced transfection with Lipofectamine 2000 reagent: primary neurons, siRNA, and high-throughput applications. Methods. 2004; 33(2):0-103.

Supplement Table

Table S 1. Designed specific siRNA sequences of TRPV4 (NM_023970.1), related to Figure 5.

	sense（5'-3'）	antisense（5'-3'）
si-1	GCGAGAUCUACCAGUACUATT	UAGUACUGGUAGAUCUCGCTT
si-2	CCGUGUCCUUCUACAUCAATT	UUGAUGUAGAAGGACACGGTT
si-3	GACGUCCAAACCUGCGUAUTT	AUACGCAGGUUUGGACGUCTT
si-NC	UUCUCCGAACGUGUCACGUTT	ACGUGACACGUUCGGAGAATT

Table S 2. 31 proteins expressed in PMVs related with calcium, related to Figure 2.

Molecular name	Molecular name
A2M	NAD^+
CCL5	SWAP70
TTR	ATP5F1A
ANXA6	ACTB
THBS1	PPBP
PPIA	SLC4A7
TGFB1	CD36
HTR2B	EVPL1
VCL	ITGB3
GSN	PLA2G7
HBA1	ITPR3
GP9	VDAC1
PECAM1	DYSF
ATP5F1B	CCR1
ITGA2B	PIK$_3$CG
TTN	

Supplement Figures and Legends

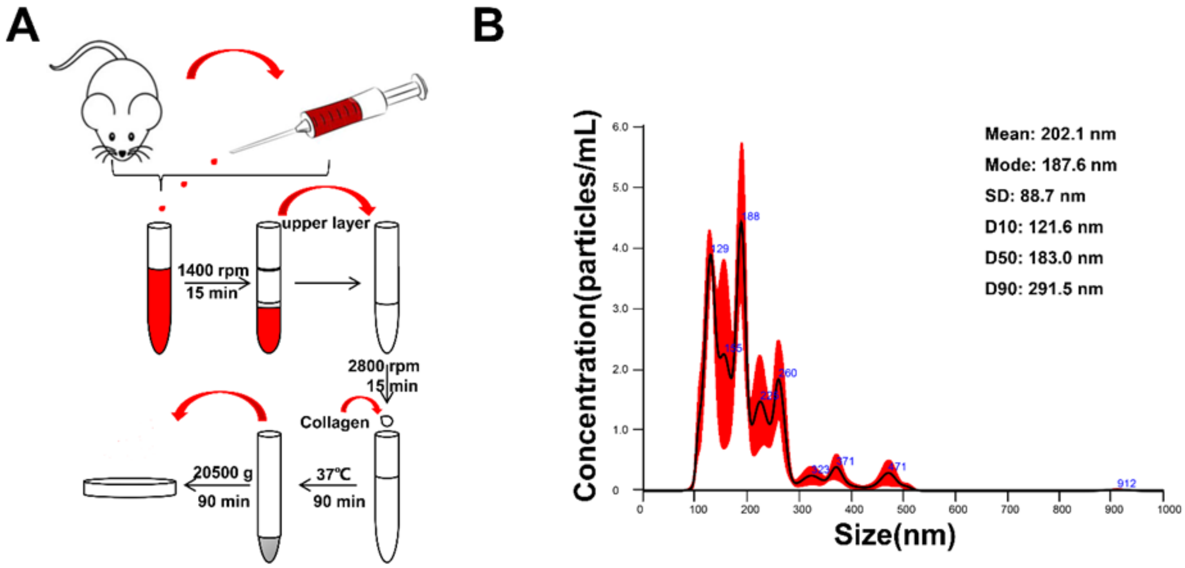

Figure S1. Procedure chart of PMVs extraction and enrichment *in vitro*(A) and PMVs size distribution detected by NanoSight3000 after collagen activation. Abscissa represents vesicles size and ordinate represents the enrichment degree of vesicles (B), related to Figure 1.

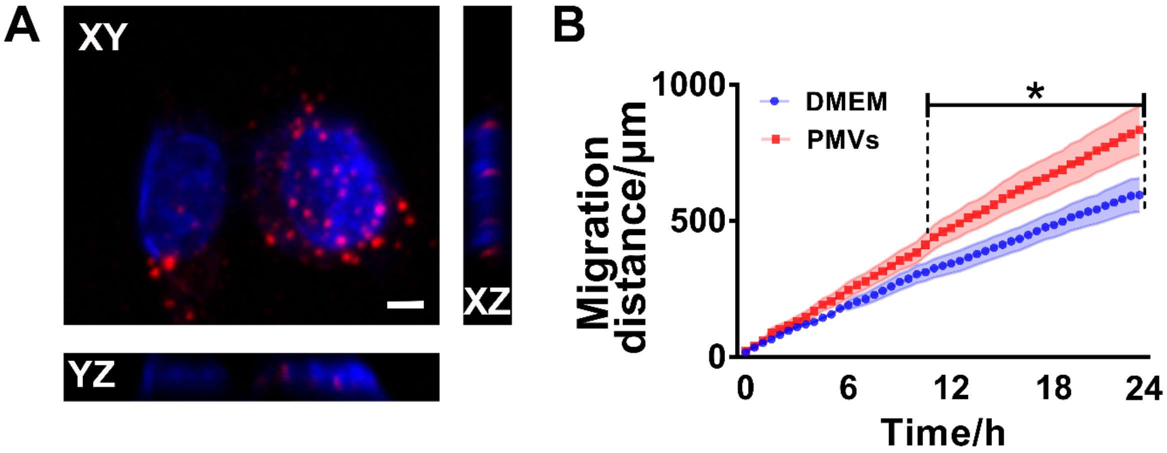

Figure S2. VSMCs were fixed and processed for staining using membrane dye PKH-26 (red), and DAPI was used as a nuclear marker, and images were collected using confocal microscopy(A). Serum-deprived VSMCs were stimulated with PMVs or DMEM for 24 h. Images were captured by EVOS FL Auto Cell Imaging system at an interval of 30 min for 24 h. The migration distance of VSMCs simulated with PMVs (red) and DMEM (blue) was shown in (B). Abscissa represents the time point, ordinate represents the migration distance and all shadow area indicated S.E.M., * $P < 0.05$, related to Figure 1.

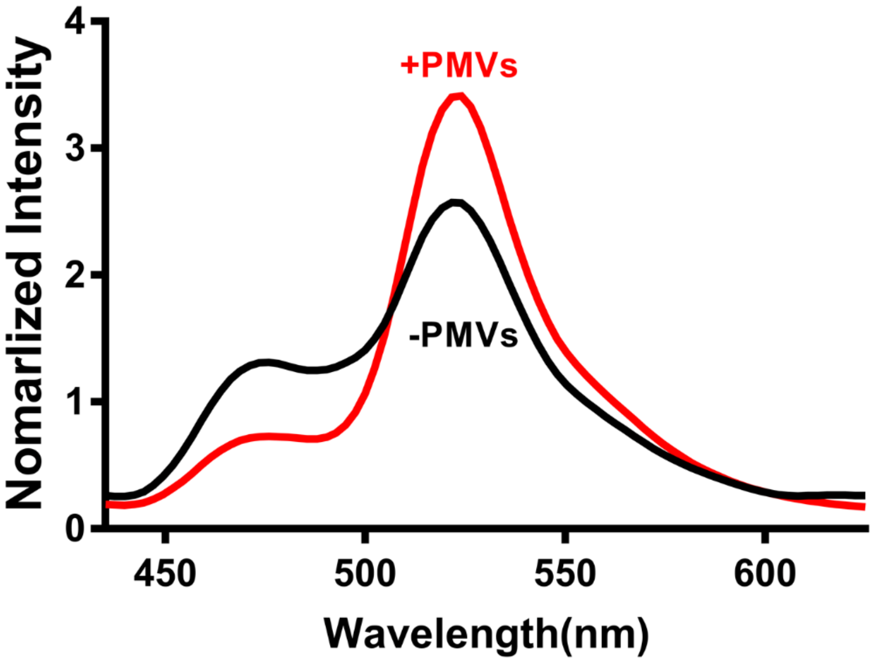

Figure S3. The emission spectra change of the cytoplasmic calcium biosensor before (black) and after (red) PMVs stimulation. related to Figure 2.

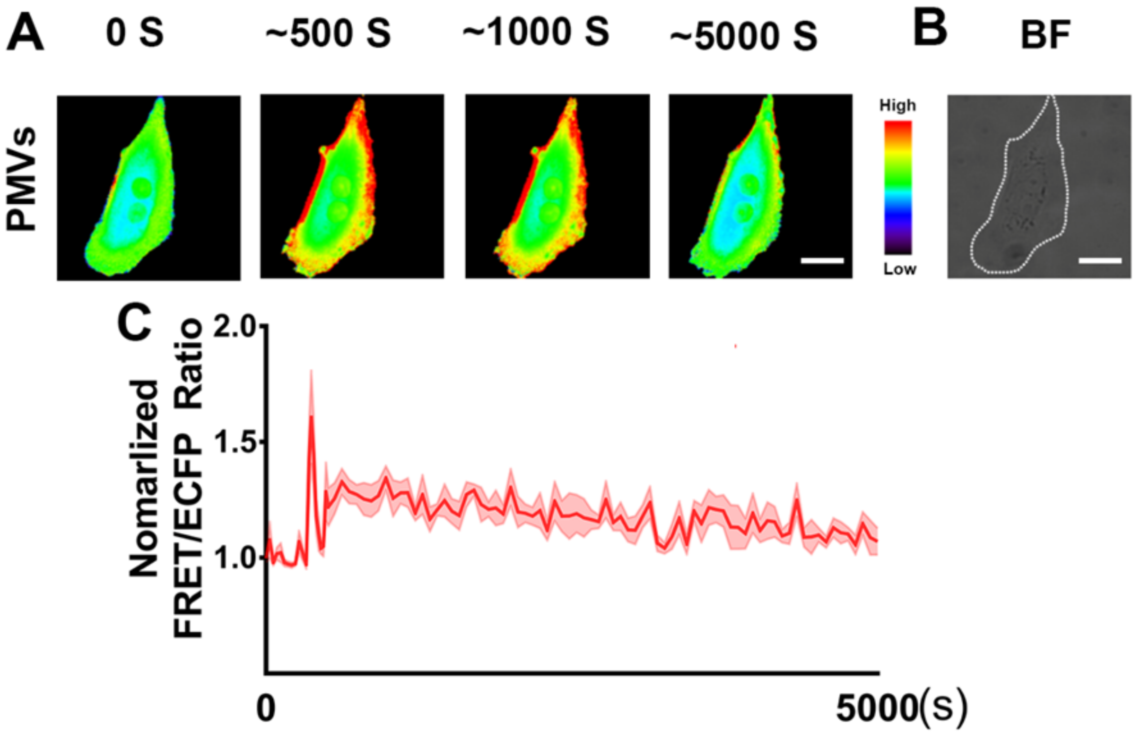

Figure S4. Representative heat maps of normalized FRET/ECFP ratio at 0 s, ~500 s, ~1000 s, ~5000 s of PMVs. The color scale bars represent the range of ratio, with cold and hot colors indicating low and high levels of calcium change (A). Bright field of the VSMC revealed that the VSMC is alive at the termination of 5000 s (B). The time courses represent the normalized FRET/ECFP ratio averaged over the cell body in the VSMC stimulated with PMVs respectively and all shadow area indicated S.E.M. (C). Scale bar: 20 μm. Data are expressed as Mean ± S.E.M.. related to Figure 2.

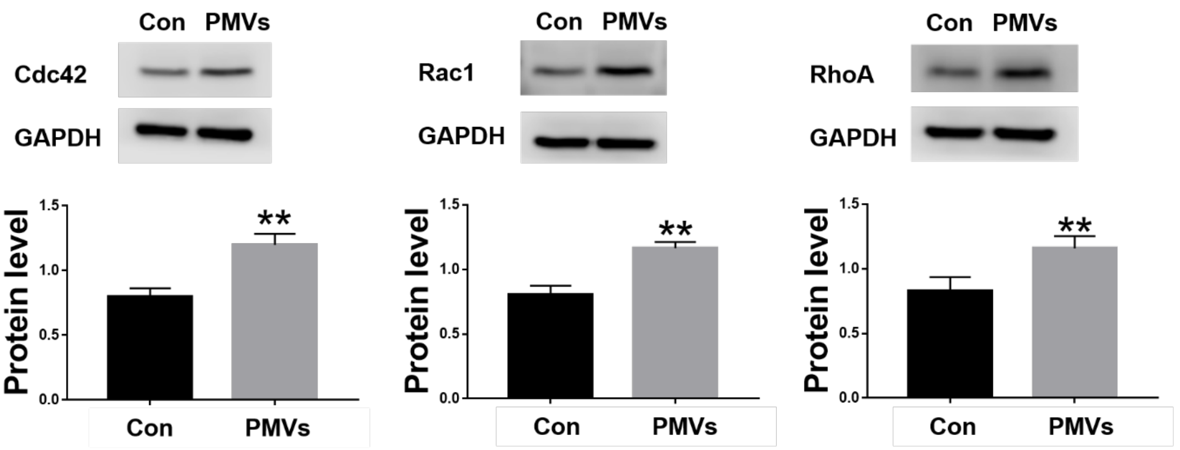

Figure S5. After 24-hour stimulation, the protein level of Cdc42, Rac1 and RhoA were significantly increased by the PMVs, which induces calcium influx in VSMCs. ** *$P < 0.01$ vs.* DMEM control, n=3.

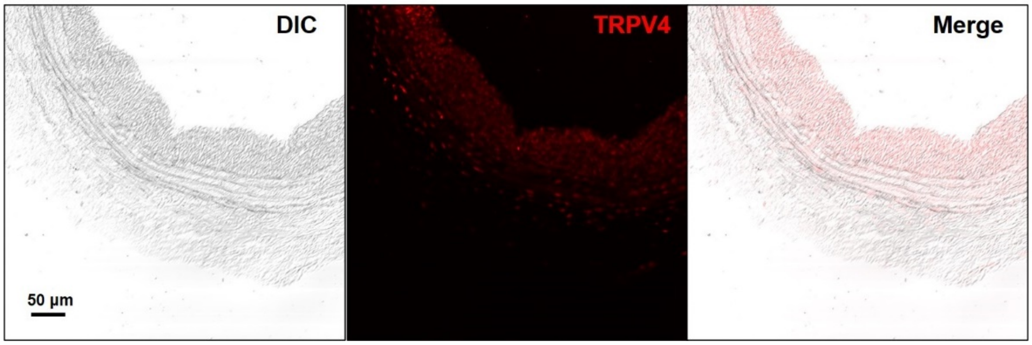

Figure S6. After the injury for 28 days, carotid arteries were collected and then TRPV4 expression (red) in the neointimal hyperplasia was detected by immunofluorescence staining. Scale bar: 50 µm.

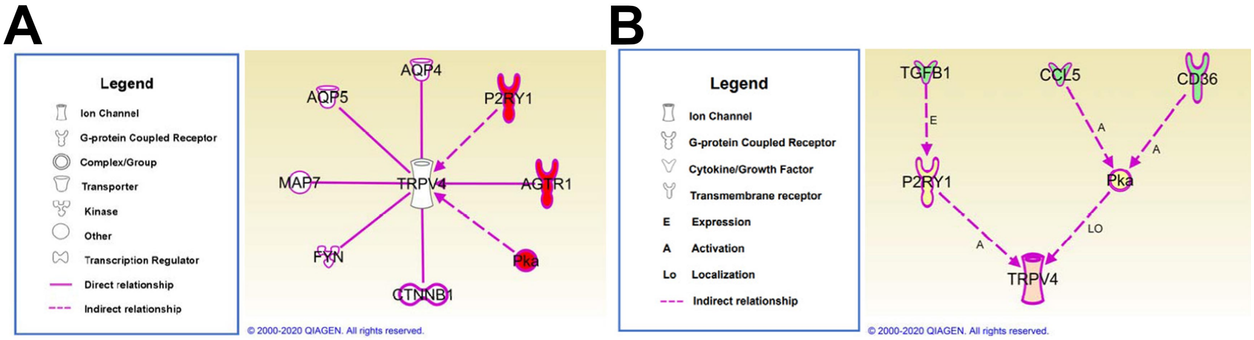

Figure S7. (A) The three upstream regulators (red) associated with TRPV4 were determined by IPA software. (B) The signaling pathways that PMVs regulated TRPV4 were determined by IPA software. Three molecules in PMVs associated with TRPV4 were indicated in green and the two upstream regulators of TRPV4 were indicated in yellow.

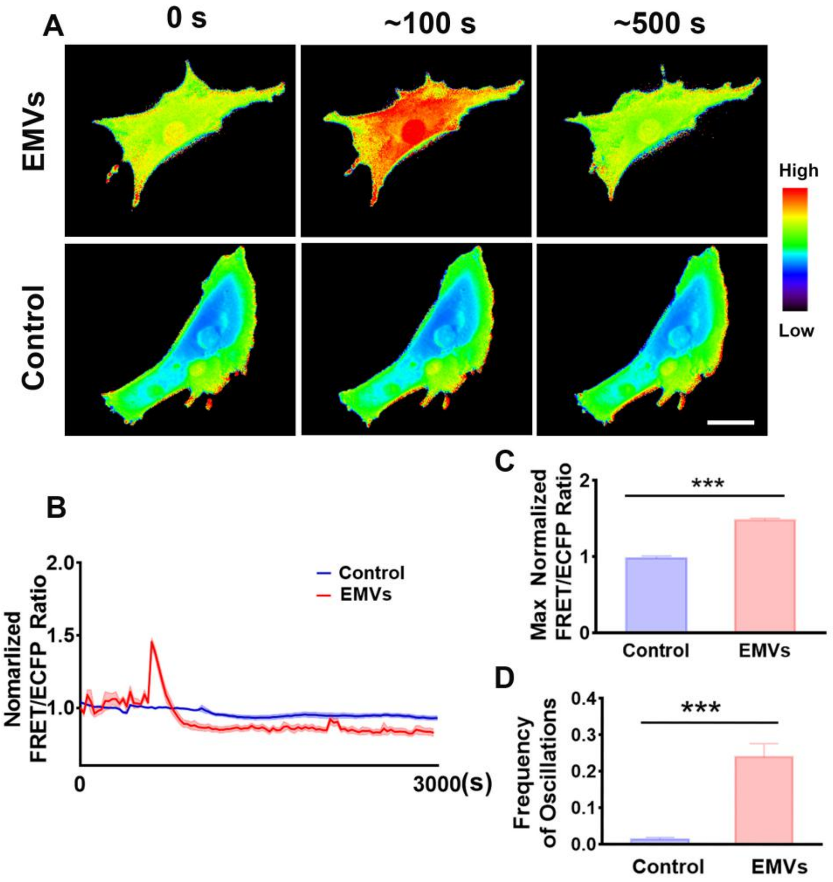

Figure S8. Calcium oscillations in VSMC in response to EMVs. (A) Time-lapsed FRET images of cytoplasmic calcium change in the VSMC treated with EMVs (top) and DMEM control (bottom). The hot and cold color represent high and low FRET rations, indicating high and low level of cytoplasmic calcium change. Scale bar: 30 μm. (B) The time courses represent the normalized FRET/ECFP ratio averaged over the cell body in the VSMC treated with EMVs (n=8) and DMEM control (n=8) and all shadow area indicated S.E.M.. Comparison of max normalized FRET/ECFP ratio (C), frequency of the cytoplasmic calcium oscillations (D) between the VSMCs treated with EMVs (n=8) and DMEM control (n=8). Data are expressed as Mean ± S.E.M.. Respectively ***P < 0.001.

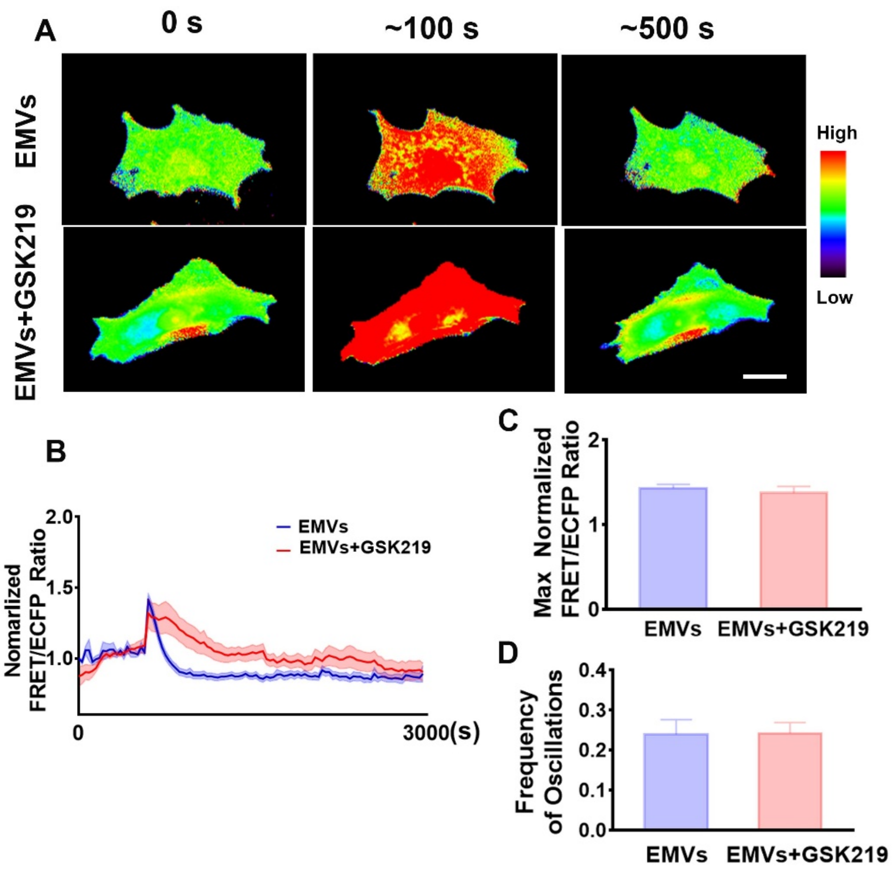

Figure S9. The role of TRPV4 antagonist GSK219 in EMVs induced calcium oscillations in VSMCs. (A) The FRET images of calcium upon EMVs application in the VSMCs pretreated with GSK219 (bottom) and DMSO control (top). The hot and cold color represent high and low FRET rations, indicating high and low level of cytoplasmic calcium change. Scale bar: 30 μm. (B) The time courses represent the normalized FRET/ECFP ratio averaged over the cell body in the VSMC pretreated with GSK219 (n=8, red) and DMSO (n=8, blue) after EMVs application and all shadow area indicated S.E.M.. Comparation of frequency of the cytoplasmic calcium oscillations (C), max normalized FRET/ECFP ratio (D) between the VSMCs pretreated with GSK219 (n=8, red) and DMSO (n=8, blue) after EMVs application.

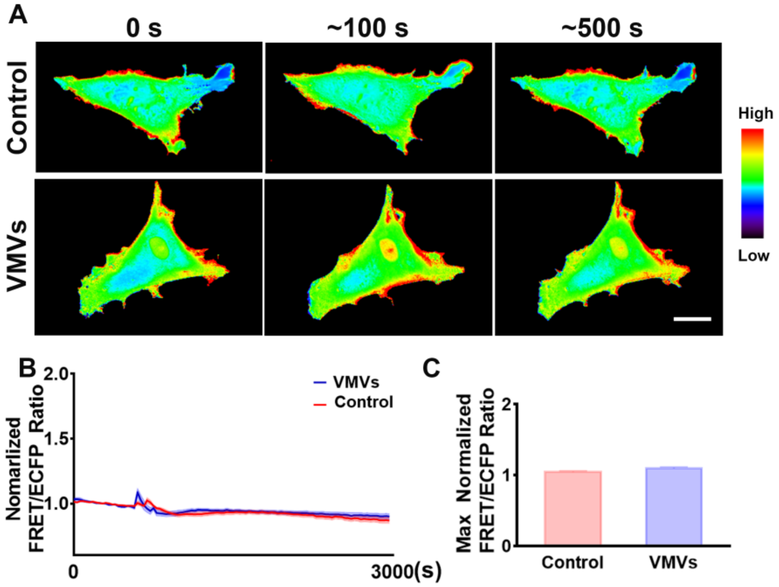

Figure S10. Calcium oscillations in VSMC in response to VMVs. (A) Time-lapsed FRET images of cytoplasmic calcium change in the VSMC treated with VMVs (top) and DMEM control (bottom). The hot and cold color represent high and low FRET rations, indicating high and low level of cytoplasmic calcium change. Scale bar: 30 µm. (B) The time courses represent the normalized FRET/ECFP ratio averaged over the cell body in the VSMC treated with VMVs (n=8) and DMEM control (n=8) and all shadow area indicated S.E.M.. (C) Comparison of max normalized FRET/ECFP ratio between the VSMCs treated with VMVs (n=8) and DMEM control (n=8). Data are expressed as Mean ± S.E.M..

Supplement Videos

Video S1 Single cell migration induced by DMEM(A) and PMVs(B), related to Figure 1.

Video S2 The FRET/ECFP ratio heat map video before and after PMVs(A) or DMEM stimulation(B), related to Figure 2.

Video S3 The FRET/ECFP ratio heat map video before and after PMVs stimulation in Ca^{2+}-free(A), 2-APB(B) and DMSO(C), related to Figure 3.

Video S4 The FRET/ECFP ratio heat map video before and after PMVs stimulation in pre-incubating in Nife(A), GSK219(B) and DMSO(C), related to Figure 4.

Video S5 The FRET/ECFP ratio heat map video before and after PMVs stimulation in si-NC(A) and si-1-cy3(B), related to Figure 5.
